# A Novel Polysaccharide from *Auricularia* *Auricula* Alleviates Thrombosis Induced by Carrageenan in Mice

**DOI:** 10.3390/molecules27154831

**Published:** 2022-07-28

**Authors:** Chun Bian, Lanyang Ji, Hang Qu, Zhenyu Wang

**Affiliations:** 1 School of Food Engineering, Harbin University, Harbin 150036, China; bianchun1980@126.com; 2 School of Chemistry and Chemical Engineering, Harbin Institute of Technology, Harbin 150006, China; 3 Heilongjiang Province Grain Quality & Safety Monitor and Technology Center, Harbin 150008, China; 2669911@126.com; 4 School of Life & Environmental Science, Wenzhou University, Wenzhou 325035, China

**Keywords:** *Auricularia auricula*, polysaccharide, structure elucidation, antithrombotic activity, thrombosis

## Abstract

The increasing incidence of cardiovascular diseases has created an urgent need for safe and effective antithrombotic agents. In this study, we aimed to elucidate the structural characteristics and antithrombotic activity of a novel polysaccharide isolated from *Auricularia* *auricula* fruiting bodies. The purified polysaccharide AAP-b2 (12.02 kDa) was composed of mannose, glucuronic acid, glucose and xylose, with a molar ratio of 89.25:30.50:4.25:1.00. Methylation and NMR analyses showed that AAP-b2 primarily consisted of →2,3)-Manp-(1→, →3)-Manp-(1→, →4)-GlcAp-(1→ and Manp-(1→. A thrombus mouse model induced by carrageenan was used in this research to evaluate its antithrombotic effect. AAP-b2 significantly inhibited platelet aggregation, reduced the black tail length and prolonged the coagulation time, including activated partial thromboplastin time (APTT), prothrombin time (PT) and thrombin time (TT), exerting a good inhibitory effect on thrombosis in mice. The antithrombotic activity of AAP-b2 was found to be related to the inhibition of platelet activation by regulation of endothelial nitric oxide synthases (eNOs), endothelin-1 (ET-1), prostacyclin (PGI2) and thromboxane B2 (TXB2), along with the enhancement of anticoagulant activity by affecting antithrombin III (AT-III) and protein C (PC) pathways.

## 1. Introduction

Cardiovascular diseases have become a critical threat to human health due to the improvement of living standards and changes of dietary patterns in recent years [1]. Thrombosis, the formation of thrombus in a blood vessel, is a main cause of cardiovascular diseases, which influence its occurrence and progression [2]. Thrombosis is a complex process, involved in platelet activation, secretion function and activation of intrinsic and extrinsic coagulation systems [3]. Currently, heparin and aspirin, the platelet activation inhibitor, are commonly used for antithrombosis [4]. However, these drugs show serious side effects in clinical practice, such as bleeding, hyperkalemia, thrombocytopenia and gastrointestinal damage [5]. Thus, effective and safe antithrombotic agents are urgently needed.

Natural polysaccharides are a group of abundant, safe and useful substances which have developed into an important source of drugs, health products and functional foods. Numerous studies have demonstrated that natural polysaccharides, derived from plants, animals and microorganisms, possess various beneficial health effects such as antioxidant, antitumor, hypoglycemic and immunomodulatory activities [6,7,8]. The antithrombotic effects of natural polysaccharides have also attracted attention recently. For instance, polysaccharides extracted from *Geoffroea spinosa* barks, blackberry (*Rubus eubatus*) seeds and red algae *Gelidiella acerosa* have been reported to have anticoagulant, antiplatelet or antithrombotic effects [9,10,11]. The biological activities of natural polysaccharides highly depend on their structural characteristics. 

*Auricularia auricula* (*A. auricula*) is an edible and medicinal fungus. It has been reported that polysaccharides from *A. auricula* show antioxidant, antitumor and radioprotective activities [12,13,14]. In a previous report, we extracted polysaccharides from the fruiting bodies of *A. auricula* (AAP) using an ultrasound-assisted method and found that the crude polysaccharide showed anticoagulant activity in vitro [15]. However, the bioactive component in AAP and its antithrombotic activity in vivo remained unclear. Therefore, in this work, AAP was further purified to isolate the bioactive component and its antithrombotic property was also evaluated by establishing a thrombus model in mice. This present study aimed to clarify the structural characteristics of an antithrombotic polysaccharide from *A. auricula* and reveal its possible action pathways. The results of this research are expected to have important implications for the development of novel potential antithrombotic agents.

## 2. Results and Discussion

### 2.1. Isolation and Purification of AAP-b2

**Figure 1:**
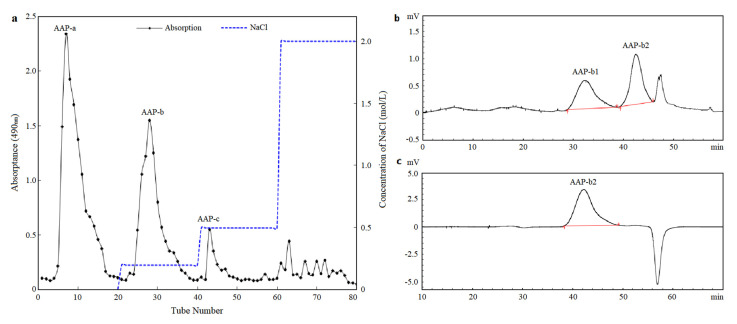
Isolation and purification of polysaccharides extracted from *A. auricula*. (**a**) Elution curve of AAP on DEAE Sepharose Fast Flow column. (**b**) HPGPC profile of AAP-b. (**c**) HPGPC profile of AAP-b2.

After purification through a DEAE Sepharose Fast Flow column, AAP was separated into three major components, namely AAP-a, AAP-b and AAP-c, with the yields of 10.34%, 25.85% and 5.52%, respectively (Figure 1a). According to the results of the hemagglutination assay in vitro (Table S1, Supplementary Material), AAP-b was the main bioactive component in AAP. However, the HPGPC spectrum indicated that AAP-b was a heterogeneous polysaccharide containing at least two main components, as shown in Figure 1b. A Superdex-200 gel filtration column was used for further purification of AAP-b, and AAP-b2 was identified to be the component responsible for the anticoagulant activity. The purity and average molecular weight of AAP-b2 were determined, and the results showed that AAP-b2 was a homogeneous polysaccharide (Figure 1c). The weight-average molecular weight (Mw) and number-average molecular weight (Mn) of AAP-b2 were 12.02 kDa and 9.77 kDa, respectively. The dispersity index (Mw/Mn) was 1.23 according to the HPGPC analysis.

### 2.2. Monosaccharide Composition of AAP-b2

**Figure 2:**
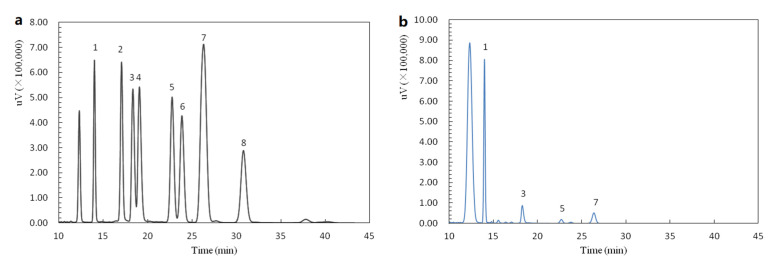
HPLC chromatograms of monosaccharide standards (**a**) and AAP-b2 (**b**). Peaks 1–8 represent mannose, rhamnose, glucuronic acid, galacturonic acid, glucose, galactose, xylose and fucose, respectively.

AAP-b2 contained 87.01 ± 1.58% carbohydrate, 1.73 ± 0.07% protein and 18.77 ± 1.25% uronic acid. The monosaccharide composition analysis indicated that AAP-b2 was mainly composed of four monosaccharides, mannose (Man), glucuronic acid (GlcA), glucose (Glc) and xylose (Xyl), with a molar ratio of 89.25:30.50:4.25:1.00 (Figure 2). These results suggested that AAP-b2 was an acidic polysaccharide, which was consistent with the previous report by Xia et al. [16]. In fact, the anticoagulant and antithrombotic activities of polysaccharides are closely related to the presence of uronic acid, based on numerous reports in the literature [17,18]. Thus, the high content of uronic acid in AAP-b2 may be an important reason for its biological activity.

### 2.3. Methylation Analysis of AAP-b2

**Figure 3:**
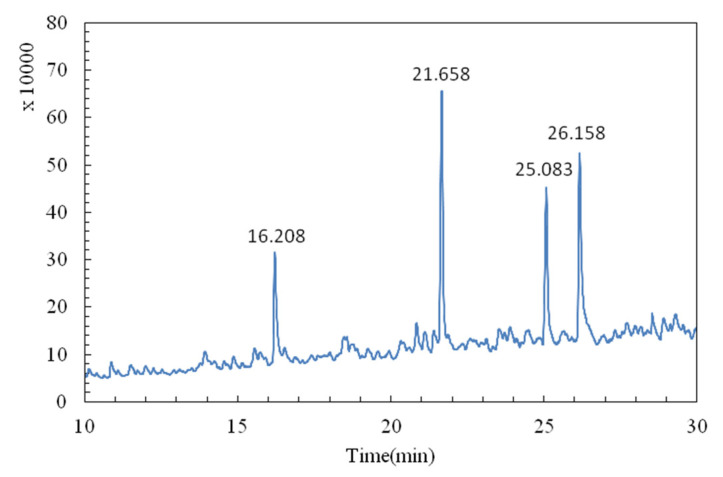
GC-MS chromatogram for methylation analysis of AAP-b2.

The glycosidic bonds in polysaccharides were investigated via methylation analysis. AAP-b2 was fully methylated and the GC-MS chromatogram showed four peaks of partially methylated alditol acetates (Figure 3). Through comparison with the CCRC spectral database, →3)-Manp-(1→, →2,3)-Manp-(1→, →4)-Glcp-(1→ and Manp-(1→ were identified. The molar ratio of these four types of glycosidic bonds was 2.1:2.0:1.5:1.0, and the mass spectra of PMAAs are presented in Figure S1. 

### 2.4. NMR Analysis of AAP-b2

**Figure 4:**
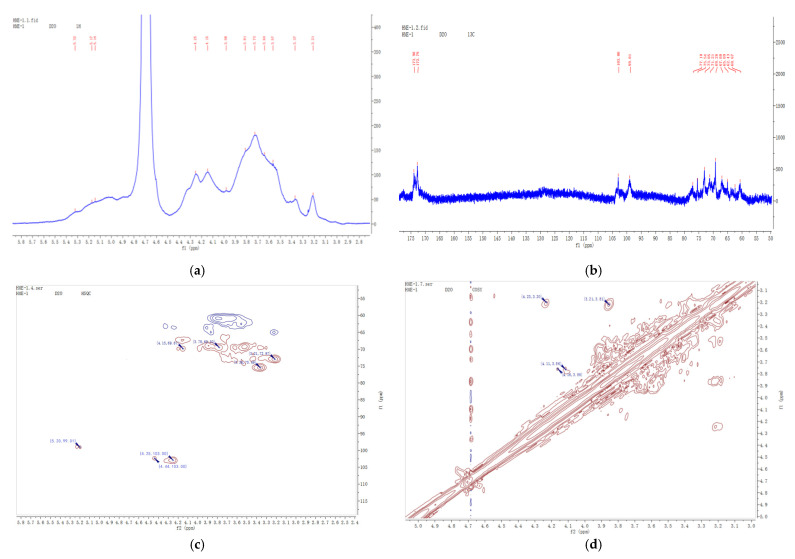
^1^H NMR (**a**), ^13^C NMR (**b**), HSQC (**c**) and COSY (**d**) spectra of AAP-b2.

1D and 2D nuclear magnetic resonance (NMR) techniques were applied to study the structure of AAP-b2. As shown in the ^1^H NMR (Figure 4a), a complex group of peaks in the spectrum was observed at δ 4.00~5.50 ppm, indicating the co-existence of α- and *β*-configuration sugar residues in AAP-b2. In the ^13^C NMR spectrum (Figure 4b), there were two obvious absorption peaks at δ 99.01 and 103.00 ppm in the anomeric carbon region (δ 95.0~110 ppm). The signals at δ 172.79/173.98 ppm, attributed to the carbonyl group, confirmed the presence of uronic acid in AAP-b2. 

Considering the structural complexity of AAP-b2, the ^1^H and ^13^C signals were further assigned according to the 2D NMR spectra and relevant literature. In the HSQC spectrum (Figure 4c), three cross-peaks of δ 4.25/103.00, 4.44/103.00 and 5.20/99.01 ppm were observed, suggesting that at least three sugar residues were present in AAP-b2. The cross-peak of δ 4.25/103.00 ppm was the typical signal of →4)-GlcAp-(1→ [19]. Based on the COSY spectrum (Figure 4d), the chemical shifts for the H-2 and H-3 of →4)-GlcAp-(1→ were δ 3.21 and 3.81 ppm, respectively. The corresponding C-2 and C-3 were δ 73.04 and 69.26 ppm by the HSQC spectrum. The H-4/C-4 were speculated to be δ 3.37 and 75.47 ppm, based on the literature [20]. The signals δ 172.79/173.98 ppm were attributed to the C-6 of →4)-GlcAp-(1→ [21]. However, the H5/C5 could not be assigned based on the current data. 

The signals δ 5.22/99.01 ppm corresponded to an α-linked residue and were assigned to H-1/C-1 of Manp-(1→ or →3)-Manp-(1→. The chemical shifts of H-2 was speculated to be δ 4.15 ppm, according to Li et al. [22]. The corresponding C-2 was δ 69.26 ppm, as shown in the HSQC spectrum. Since there was a cross-peak between δ 4.15 and 3.86 ppm in the COSY spectrum, the H-3 was δ 3.86 ppm, and the corresponding C-3 was δ 71.21 ppm, which belonged to the residue Manp-(1→. Meanwhile, the signal at δ 3.93/77.26 ppm was assigned to the H-3/C-3 of →3)-Manp-(1→. As both δ 3.93 and 3.85 ppm showed a weak cross with δ 3.64 ppm in the COSY spectrum, it was speculated that δ 3.64 ppm belonged to the H-4 of Manp-(1→ and →3)-Manp-(1→. The corresponding C-4 was δ 69.26 ppm. The signals δ 3.86/73.04 ppm and δ 3.53–3.82/60.67 ppm were assigned to the H-5/C-5 and H-6/C-6, respectively, according to Xu et al. [23]. Similarly, the signals δ 4.44/103.00 ppm corresponded to a *β*-linked residue and were assigned to the H-1/C-1 of →2,3)-Manp-(1→. Combining the NMR spectra and reported literature, the chemical shifts of →2,3)-Manp-(1→ were δ 3.49, 3.64, 3.73, 3.91 and 4.05 ppm for H-2, H-3, H-4, H-5 and H-6, respectively. The corresponding C-2, C-3, C-4, C-5 and C-6 were assigned to δ 71.07, 72.97, 66.82, 69.16 and 60.66 ppm. These results were consistent with the previous report by You et al. [24].

The present NMR results validated the monosaccharide composition and methylation analysis of AAP-b2. However, the molecular structure of AAP-b2 could not be comprehensively identified due to the insufficient useful information from the HMBC spectrum. Overall, AAP-b2 is mainly composed of four glycosidic bonds and the bone structure may be the connection of →2,3)-Manp-(1→, →3)-Manp-(1→ →4)-GlcAp-(1→and Manp-(1→.

### 2.5. Effects of AAP-b2 on Platelet Aggregation In Vitro

**Figure 5:**
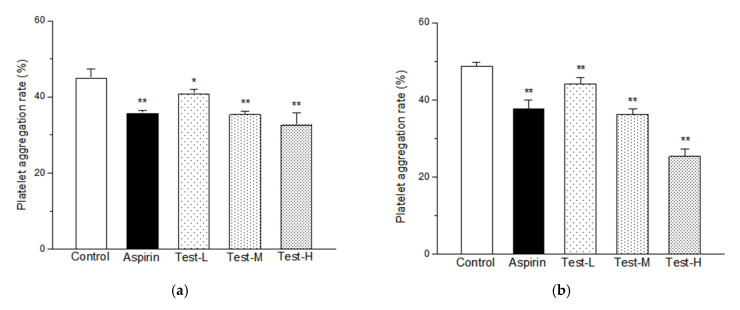
The effects of AAP-b2 on ADP- (**a**) and collagen-induced (**b**) platelet aggregation in vitro. * *p* < 0.05 and ** *p* < 0.01 compared to the ADP or collagen control groups.

Agonists including adenosine diphosphate (ADP) and collagen were used to evaluate the effects of AAP-b2 on in vitro platelet aggregation. As presented in Figure 5, AAP-b2 significantly inhibited ADP- and collagen-induced platelet aggregation (*p* < 0.05). The high dose of AAP-b2 (50 μg/mL) inhibited ADP- and collagen-induced platelet aggregation by 48.03% and 27.44%, respectively. These results suggested that AAP-b2 has the potential to inhibit platelet aggregation induced by multiple platelet agonisists, which may play an important role in the antithrombotic activity of AAP-b2.

### 2.6. Effects of AAP-b2 on Thrombus Length

**Figure 6:**
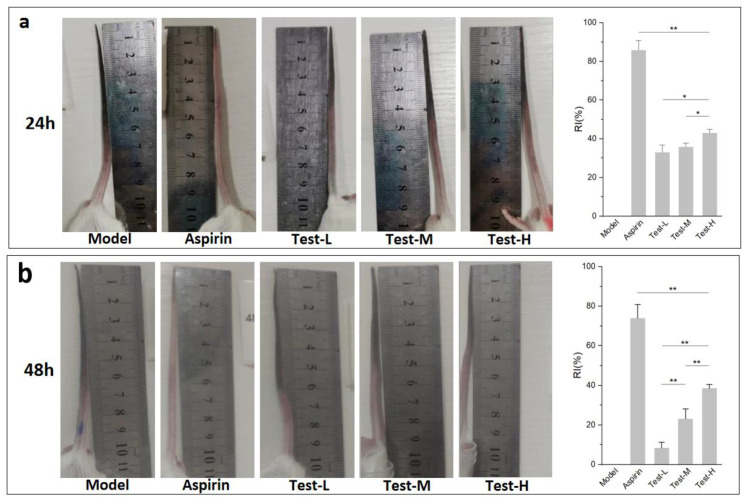

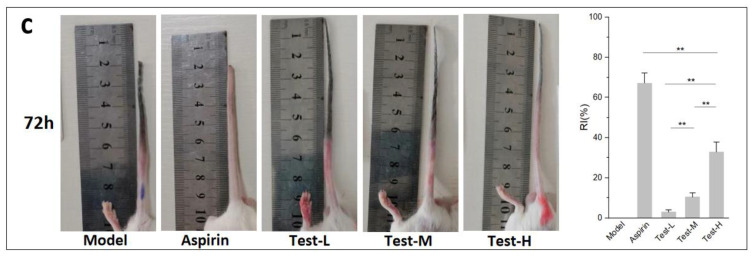
Effects of AAP-b2 on black tail length of mice and the inhibition rate of thrombus relative length (IR) at 24 h (**a**), 48 h (**b**) and 72 h (**c**) after modeling by carrageenan. * *p* < 0.05 and ** *p* < 0.01.

Carrageenan is a commonly used inducer of thrombosis. After intraperitoneal injection of carrageenan, thrombosis occurs in a mouse’s tail, and this change is visible and easy to measure. As shown in Figure 6, the mice tails turned black from the tip after modeling, indicating the formation of thrombi within the blood vessels of the tails. The black tail length became larger and the boundary of the thrombus in mice tails became clearer with the extension of time (from 24 h to 72 h). After 72 h, some mice tails even broke off due to the thrombosis (Figure 6c). The black tail length of each treatment group at 24 h, 48 h and 72 h after modeling was determined to calculate the inhibition rate of the thrombus relative length (IR), respectively. It could be found that AAP-b2 inhibited the thrombosis in mice tails and the effect presented a significant dose-dependent manner after 48 h (*p* < 0.01). The IR of high-dose AAP-b2 in our study was as high as 42.86 ± 1.97%, which was superior to the results reported for bioactive substances with antithrombotic activity, such as the extracts of *Rehmanniae* *radix* [25], *Pseudocedrela kotschyi* and *Adenia cissampeloides* [26].

### 2.7. Effects of AAP-b2 on Coagulation Parameter

The effects of AAP-b2 on coagulation parameters in carrageenan-treated mice were evaluated and the results are shown in Table 1. Compared with the model group, high-dose AAP-b2 markedly prolonged the coagulation time, including activated partial thromboplastin time (APTT) (*p* < 0.01), prothrombin time (PT) (*p* < 0.05) and thrombin time (TT) (*p* < 0.01), and reduced the content of fibrinogen (FIB) (*p* < 0.01). APTT and PT are related to the intrinsic and extrinsic coagulation pathways, respectively [27]. TT is an important index reflecting the conversion from fibrinogen into fibrin [28]. The declined FIB in blood is responsible for reducing blood viscosity, suppressing red platelet and blood cell aggregation and finally resulting in the inhibition of thrombosis [25]. Thus, our results indicated that AAP-b2 exerted anticoagulant activity by inhibiting coagulation pathways and hindering fibrin formation in mice.

### 2.8. Histological Examination

**Figure 7:**
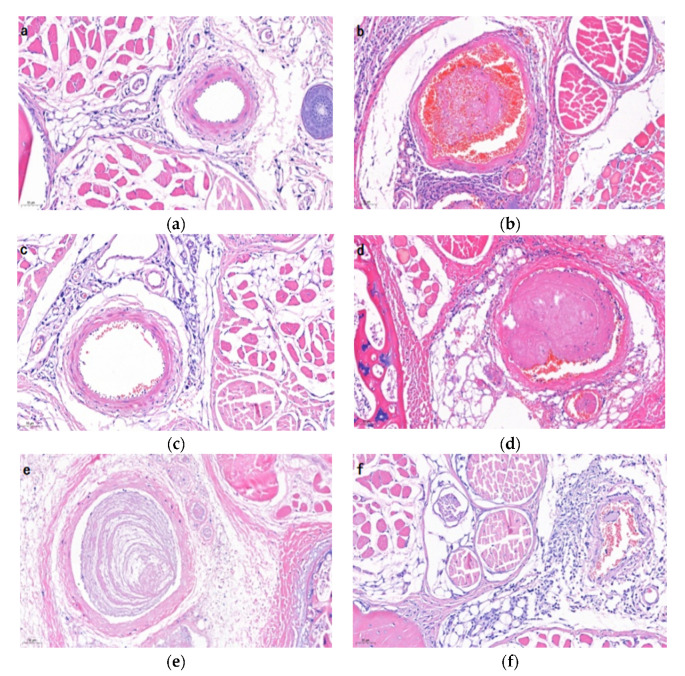
Effects of AAP-b2 on the histomorphology of tail tissues in thrombosis model mice. (**a**) Control group, (**b**) model group, (**c**) aspirin group, (**d**) Test-L group, (**e**) Test-M group, (**f**) Test-H group.

The hematoxylin and eosin (H&E) staining of mice tail tissues are shown in Figure 7. The vascular structure in the control group was clear and without thrombi (Figure 7a). However, different degrees of thrombosis were observed in the carrageenan treatment groups (Figure 7b–f). As seen in Figure 6b, the occlusive thrombus occupied almost the entire vessel and there was a large amount of closely cross-linked fibrin in the thrombosis. This result was consistent with the previous report [29]. Compared with the model group, middle and high dose of AAP-b2 exerted inhibitory effects on thrombosis in mice tails. Approximately 75% of the vessel was blocked by thrombus in the middle-dose group, but the thrombus was nonobstructive due to the loosely cross-linked fibrin. Meanwhile, only a small amount of thrombus was formed in the vessel and there was no obvious blockage in the high-dose group. The H&E staining results provided strong evidence that AAP-b2 has potential to suppress thrombosis. 

### 2.9. Antithrombotic Pathway Analysis of AAP-b2

#### 2.9.1. Effect of AAP-b2 on Platelet Activation in Mice

**Figure 8:**
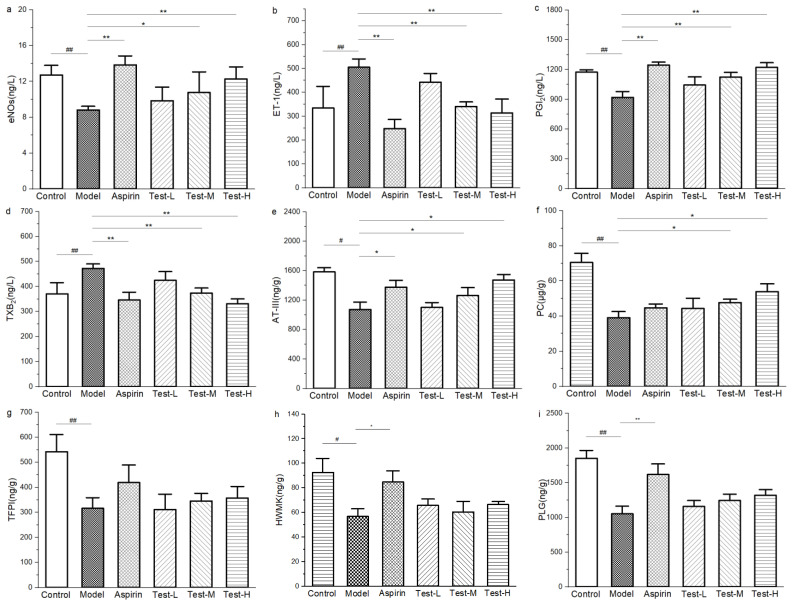
Effects of AAP-b2 on nitric oxide synthases (eNOs) (**a**), endothelin-1 (ET-1) (**b**), prostacyclin (PGI2) (**c**), thromboxane B2 (TXB2) (**d**), antithrombin III (AT-III) (**e**), protein C (PC) (**f**), tissue factor pathway inhibitor (TFPI) (**g**), high molecular weight kininogen (HMWK) (**h**) and plasminogen (PLG) (**i**) in thrombosis model mice. ^#^ *p* < 0.05 and ^##^ *p* < 0.01 vs. control group. * *p* < 0.05 and ** *p* < 0.01 vs. model group.

Vascular endothelial tissue can secrete a series of antithrombotic substances, some of which participate in inhibition of platelet activation and thrombus formation. In this study, serum endothelial nitric oxide synthases (eNOs), endothelin-1 (ET-1), prostacyclin (PGI2) and thromboxane B2 (TXB2) were determined in order to evaluate the effect of AAP-b2 on platelet activation (Figure 8). As shown in Figure 8a,b, carrageenan treatment caused a sharp decrease in eNOs (*p* < 0.01) and increase in ET-1 (*p* < 0.01), indicating vascular endothelial dysfunction [30]. AAP-b2 reversed this trend in a dose-dependent manner. Compared to the model group, the serum eNOs was markedly elevated from 8.81 ± 0.41 ng/L to 12.27 ± 1.35 ng/L in the high-dose AAP-b2 treatment group (Figure 7a, *p* < 0.01). Moreover, the level of ET-1 was significantly declined to 313.98 ± 58.09 ng/L (Figure 8b, *p* < 0.01). The increased eNOs had potential to promote the generation of nitric oxide (NO) and then upregulate cGMP protein kinase G (PKG) signaling, thereby inhibiting platelet aggregation [31]. 

PGI2 and thromboxane A2 (TXA2), a pair of common prostanoids, are also crucial factors in the vascular system. These two factors play opposite roles in regulation of vascular homeostasis. In detail, PGI2 is able to suppress platelet aggregation, and TXA2 exerts a procoagulant effect to the contrary [32]. Since TXA2 is extremely unstable and rapidly metabolized to TXB2, TXB2 is often used to reflect the level of TXA2 [33]. As presented in Figure 8c,d, the serum PGI2 was evidently decreased (*p* < 0.01) and TXB2 was significantly increased (*p* < 0.01) due to the damage of endothelial cells induced by carrageenan [34]. However, there was an apparent augment of PGI2 in the middle- and high-dose AAP-b2 treatment groups (Figure 8c, *p* < 0.01). Meanwhile, the level of serum TXB2 was dramatically reduced compared with the model group (Figure 8d, *p* < 0.01). Elevated PGI2 tends to inhibit platelet activation through regulation of cAMP-mediated signaling by enhancing cAMP protein kinase A (PKA) activity, which probably plays a key role in the antithrombotic activity of APP-b2 [35]. These findings suggested that the inhibition of platelet activation may be involved in the protection of AAP-b2 against vascular injury in model mice.

#### 2.9.2. Effect of AAP-b2 on Anticoagulant Activity

It is well known that anticoagulant activity is closely related to thrombosis. The activated procoagulant factors are regulated by the anticoagulant factors or pathways, including antithrombin III (AT-III), protein C (PC) and tissue factor pathway inhibitor (TFPI) [36]. AT-III is an important endogenous anticoagulant which can inhibit the activities of thrombin and coagulation factors. Our study showed that the AT-III level in the model group was markedly decreased compared with the control group (Figure 8e, *p* < 0.05). Middle and high doses of AAP-b2 effectively upregulated the content of AT-III (*p* < 0.05), but there was no significant difference in the low-dose group (*p* > 0.05). The elevated AT-III level represented the enhanced anticoagulant activity, thereby inhibiting thrombus formation in mice. PC, a major regulator for coagulation, can be activated by the thrombin–thrombomodulin complex [37]. After treatment with carrageenan, PC was at an obviously low level in comparison with the untreated control (Figure 8f, *p* < 0.01). Interestingly, an evident increase in PC was observed in the middle- and high-dose AAP-b2 groups (*p* < 0.05), but not in the positive group. These results indicated that AAP-b2 may delay thrombosis by enhancing the synthesis of PC and improving the anticoagulant activity in mice. In addition, TFPI, produced by endothelial cells of the microvasculature, is a natural regulator of extrinsic blood coagulation. Decreased TFPI usually reflects an endothelial dysfunction with damaged synthesis and secretion of TFPI [37]. As exhibited in Figure 8g, TFPI was sharply reduced in the model group (*p* < 0.01). Neither aspirin nor AAP-b2 reversed the decline of TFPI in mouse livers (*p* > 0.05), suggesting that AAP-b2 did not affect coagulation activity and thrombosis in mice by inhibiting the synthesis of tissue factor. Thus, AAP-b2 has potential to enhance anticoagulant activity by increasing AT-III and PC, and thereby to inhibit thrombosis in mice.

#### 2.9.3. Effect of AAP-b2 on Fibrinolytic Capacity

Plasminogen (PLG) can be converted into fibrinolys under the action of an activator. And the fibrinolys tends to degrade fibrin and other proteins. Therefore, PLG directly affects the fibrinolytic capacity. Enhanced fibrinolytic activity is beneficial to the degradation of thrombi [38]. High-molecular-weight kininogen (HMWK) is a key activator for the fibrinolytic system and a commonly used indicator for evaluating fibrinolytic capacity. As presented in Figure 8h,i, compared with the control group, HMWK and PLG sharply dropped in the model group (*p* < 0.01), denoting a decline in fibrinolytic activity. However, AAP-b2 had no significant effect on the levels of PLG and HMWK (*p* > 0.05). These results implied that AAP-b2 failed to exert antithrombotic activity by improving the fibrinolytic capacity of mice.

## 3. Materials and Methods

### 3.1. Materials and Reagents

Dried *A.* *auricula* fruiting bodies were collected from Yichun (Heilongjiang, China) and authenticated by Prof. Zhenyu Wang of Harbin Institute of Technology. The DEAE Sepharose Fast Flow column was obtained from Qianchun Bio Co. (Yancheng, Jiangsu, China). Superdex-200 gel was provided by Borui Saccharide Biotech Co. (Yangzhou, Jiangsu, China). Monosaccharide standards and HPLC-grade solvents were purchased from Sigma-Aldrich Chemical Co. (St, Louis, MO, USA). ADP and collagen were purchased from Chrono-Log Co. (Havertown, PA, USA). FIB, APTT, PT and TT Assay Kits were provided by Sysmex Co. (Shanghai, China). eNOs, PGI2, TXB2 and PLG Assay Kits were obtained from Nanjing Jiancheng Bioengineering Institute (Nanjing, Jiangsu, China). ET-1, AT-III, PC, TFPI and HMWK Assay Kits were purchased from Jingmei Biotechnology (Yancheng, Jiangsu, China). All the other reagents were of analytical grade.

### 3.2. Extraction and Purification of Polysaccharides

AAP was extracted according to the previously described method [15]. A total of 0.4 g AAP was dissolved in 2.0 mL distilled water and loaded onto a DEAE Sepharose Fast Flow column (Φ 2.6 cm × 20 cm). The column was sequentially eluted with distilled water, 0.2, 0.5 and 2.0 mol/L NaCl solutions at a flow rate of 1.0 mL/min. The fraction eluted by 0.2 mol/L NaCl solution (AAP-b) with the strongest anticoagulant activity was collected for further purification by a Superdex-200 gel filtration column (Φ 2.6 cm × 20 cm), which was then eluted with distilled water at a flow rate of 0.8 mL/min. Two fractions, AAP-b1 and AAP-b2, were obtained, of which AAP-b2, as the main component responsible for the anticoagulant activity, was enriched for further study.

### 3.3. Structure Elucidation

#### 3.3.1. Molecular Weight Determination

The average molecular weight of AAP-b2 was determined using high-performance gel permeation chromatography (LC-10A, Shimadzu, Kyoto, Japan) with a refractive index detector (RID-20A). Three gel chromatography columns, BRT105, BRT104 and BRT102 (Φ 8.0 mm × 300 mm), were connected in series. Sodium chloride (0.05 mol/L) was used as the mobile phase and the flow rate was 0.6 mL/min. Commercial dextrans with molecular weights ranging from 1.15 kDa to 409.8 kDa were chosen as standard polysaccharides to prepare the calibration curve, and the average molecular weight of AAP-b2 was calculated using its retention time.

#### 3.3.2. Monosaccharide Composition Determination

The monosaccharide composition of AAP-b2 was determined via the method of high-performance liquid chromatography. The detailed operation process was as described in the previous report [15].

#### 3.3.3. Methylation Analysis

AAP-b2 was methylated and then hydrolyzed in 1 mL of trifluoroacetic acid (TFA, 2 mol/L) at 110 °C for 1.5 h [39]. The excess TFA in hydrolysate was removed by evaporation under reduced pressure. The dry residues were reduced using 60 mg of sodium borohydride for 8 h and acetic acid was added to neutralize the solution. Afterwards, acetic anhydride was added and the reaction was kept at 100 °C for 1 h. Toluene was added to remove the excess acetic anhydride. The resulting product was redissolved in trichloromethane, washed with ultrapure water and evaporated by rotation. Finally, the product was dissolved in trichloromethane and analyzed using a gas chromatography-mass spectrometry system (GC-MS, QP2010, Shimadzu, Kyoto, Japan) equipped with a RXI-5 SIL column (30 m × 0.25 mm × 0.25 mm). The temperature was increased from 120 °C to 250 °C at a rate of 3 °C/min and maintained for 5 min. The carrier gas was helium, and the flow rate was 1 mL/min.

#### 3.3.4. NMR Spectroscopy Analysis

First, 30 mg of AAP-b2 was dissolved in 1 mL of deuterium oxide. The 1D NMR (^1^H and ^13^C) and 2D NMR (HSQC and COSY) spectra were recorded using a Bruker Avance III-500 spectrometer (Bruker, Rheinstetten, Germany) equipped with a cryogenic probe (Bruker, Prodigy BBO 500 S1).

### 3.4. Animal Experiments

#### 3.4.1. Animals and Treatments

Male SPF Kunming mice were housed in a pathogen-free animal laboratory (temperature: 25 ± 2 °C, humidity: 55%~65%, 12 h light-dark cycle) and had free access to disinfected feed and clean water. After acclimatization to laboratory conditions for 5 d, all mice were divided into six groups (*n* = 16 per group). The detailed grouping was as shown in Table 2. Animal guidelines and protocols were in compliance with the Institutional Animal Ethics Committee of Harbin University, China. All mice were given an equal volume of saline or aspirin or AAP-b2 solution daily. The polysaccharide and aspirin solutions used in this research were freshly prepared. After 14 d, all groups except the control group were injected intraperitoneally with 0.5% carrageenan (type I) solution to establish a thrombus model [40]. After modeling, all mice were continuously raised for another 3 d. Blood samples were collected from each group. The mice were sacrificed, and the liver tissues were dissected and stored at −80 °C for further assays.

#### 3.4.2. Platelet aggregation

ADP or collagen were used to induce platelet aggregation in vitro. The platelet-rich and platelet-poor plasma were prepared according to the previous report [41]. Then, saline, aspirin or AAP-b2 (12.5, 25 and 50 μg/mL) were added, followed by stimulation with ADP (10 μM) or collagen (15 μg/mL). The platelet aggregation was quantified based on the formula: Platelet aggregation rate (%) = (1 − OD_1_/OD_2_) × 100%, where OD_1_ represents the absorbance of the sample after stimulation by ADP or collagen, and OD_2_ represents the absorbance of the sample before stimulation by ADP or collagen.

#### 3.4.3. Determination of Thrombus Length

The full length of the mouse tail (L1) and the black tail length (L2) were measured using a caliper after modeling for 24 h, 48 h and 72 h, respectively. The relative average tail length (RATL) was calculated following the formula: RATL (%) = L2/L1 × 100%. The inhibition rate of thrombus relative length (IR) in each group was calculated based on the formula: IR(%) = (1 − RATL2/RATL1) × 100%, where RATL1 and RATL2 represent the average RATL values in the model group and test group, respectively.

#### 3.4.4. Measurement of APTT, PT, TT and FIB 

Mouse blood was collected 2 h after injection with carrageenan. First, 250 μL of plasma supernatant from each group was used to prepare platelet-poor plasma [1]. Then, APTT, PT, TT and FIB were determined using the corresponding assay kits strictly according to the manufacturer’s instructions.

#### 3.4.5. Histological Examination

The same parts of the mouse tails in each group were fixed with 10% formalin, embedded in paraffin, cut into pieces and stained with hematoxylin and eosin. The histological changes in mice tails were observed using a light microscope (Olympus CK31, Olympus, Japan).

#### 3.4.6. Enzyme-linked Immunosorbent Assay

An enzyme-linked immunosorbent assay (Elisa) was applied to analyze the antithrombotic pathway of AAP-b2. Serum eNOs, ET-1, PGI2 and TXB2 were determined using assay kits to analyze the effect of AAP-b2 on platelet activation in mice. AT-III, PC and TFPI in mouse livers were measured to assess the anticoagulant effects of AAP-b2. Additionally, HMWK and PLG were detected to evaluate the effect of AAP-b2 on fibrinolytic activity in mice.

### 3.5. Statistical Analysis

All experiments in this study were performed in triplicate. The data were presented as mean ± standard deviation and the statistical difference was analyzed using one-way analysis of variance by SPSS software (version 17.0). Differences were considered significant at *p* < 0.05 and *p* < 0.01.

## 4. Conclusions

A novel polysaccharide AAP-b2 with an average molecular weight of 12.02 kDa was isolated and purified from *A. auricula* fruiting bodies. The structural analysis showed that AAP-b2 consisted of mannose, glucuronide, glucose and xylose, and its main chain contained →2,3)-Manp-(1→, →3)-Manp-(1→, →4)-GlcAp-(1→ and Manp-(1→. The in vivo antithrombotic activity of AAP-b2 was studied by establishing a thrombus mouse model. AAP-b2 had potential to inhibit thrombosis and the action mechanism was related to the inhibition of platelet activation and the enhancement of anticoagulant activity.

## Figures and Tables

**Table 1:** Effects of AAP-b2 on activated partial thromboplastin time (APTT), prothrombin time (PT), thrombin time (TT) and fibrinogen (FIB) in thrombus mouse model.

Groups	APTT (s)	PT (s)	TT (s)	FIB (mg/mL)
Control	35.86 ± 0.05	17.60 ± 0.29	21.07 ± 0.48	1.86 ± 0.03
Model	30.67 ± 0.37 ^##^	13.37 ± 0.34 ^#^	16.90 ± 0.29 ^##^	3.50 ± 0.06 ^##^
Aspirin	34.63 ± 0.09 **	16.27 ± 0.37 **	19.77 ± 0.57 **	2.49 ± 0.02 **
Test-L	30.87 ± 0.12	12.70 ± 0.22	16.80 ± 0.29	3.50 ± 0.07
Test-M	31.30 ± 0.37	12.77 ± 0.25	18.03 ± 0.59	3.24 ± 0.02 **
Test-H	32.57 ± 0.29 **	14.00 ± 0.22 *	18.73 ± 0.37 **	2.70 ± 0.02 **

^#^*p* < 0.05 and ^##^ *p* < 0.01 vs. control group. * *p* < 0.05 and ** *p* < 0.01 vs. model group.

**Table 2:** Groups of experimental animals.

Groups	Experimental Treatments			
	Saline	AAP-b2	Aspirin	Carrageenan (0.5%)
Control	^a^	-	-	-
Model	^a^	-	-	10 mL/kg b.w.
Aspirin	-	-	20 mg/kg b.w.	10 mL/kg b.w.
Test-L	-	50 mg/kg b.w.	-	10 mL/kg b.w.
Test-M	-	100 mg/kg b.w.	-	10 mL/kg b.w.
Test-H	-	200 mg/kg b.w.	-	10 mL/kg b.w.

^a^ The volumes of saline in the control and model groups were as the same as those of other groups. “-” represents that the reagent was not used in this group. “b.w.” is the abbreviation of body weight.

## Data Availability

Not applicable.

## Supplementary Materials

The following supporting information can be downloaded at: https://www.mdpi.com/article/10.3390/molecules27154831/s1, Figure S1: Mass spectra scanning of glycoside linkages of Manp-(1→ (a), →3)-Manp-(1→ (b), →4)-Glcp-(1→ (c) and →2,3)-Manp-(1→ (d); Table S1: Effects of AAPs on FIB, APTT, PT and TT in vitro.

## Sample Availability Statement

Samples of the compounds are available from the authors.
