# Supplementary material for: A Novel Polysaccharide from Auricularia Auricula Alleviates Thrombosis Induced by Carrageenan in Mice
Source: Molecules. 2022 Jul 28;27(15):4831. doi: 10.3390/molecules27154831 (PMC9369961; doi:10.3390/molecules27154831)
Supplement: Supplementary file 1 [file molecules-27-04831-s001.zip › molecules-1823974-supplementary.pdf]

**Table S1.** Effects of AAPs on FIB, APTT, PT and TT in vitro

| Sample | Concentration (μg/mL) | Clotting time (s) | | |
|---|---|---|---|---|
| | | APTT | PT | TT |
| *Saline* | - | 28.0 ± 0.2 | 11.3 ± 0.1 | 17.7 ± 0.1 |
| *Heparin* | 2.5 IU/mL | 30.3 ± 0.1** | 13.6 ± 0.5** | 21.4 ± 0.1** |
| | 25 IU/mL | > 200 | > 200 | > 200 |
| *AAP* | 12.5 | 28.9 ± 0.1** | 11.8 ± 0.1 | 18.0 ± 0.1* |
| | 25 | 31.3 ± 0.3** | 11.7 ± 0.1 | 22.3 ± 0.3** |
| | 50 | 31.9 ± 0.2** | 11.0 ± 0.2 | 25.3 ± 0.3** |
| *AAP-a* | 12.5 | 28.3 ± 0.1 | 11.4 ± 0.2 | 16.5 ± 0.2 |
| | 25 | 28.4 ± 0.1 | 11.6 ± 0.1 | 16.3 ± 0.1 |
| | 50 | 28.4 ± 0.3 | 11.5 ± 0.2 | 16.6 ± 0.1 |
| *AAP-b* | 12.5 | 36.0 ± 0.1** | 11.4 ± 0.2 | 33.7 ± 0.2** |
| | 25 | 48.4 ± 0.3** | 12.6 ± 0.2** | 42.4 ± 0.2** |
| | 50 | 64.5 ± 0.4** | 13.7 ± 0.3** | 62.7 ± 0.3** |
| *AAP-c* | 12.5 | 26.2 ± 0.1 | 16.2 ± 0.2** | 20.3 ± 0.1** |
| | 25 | 27.5 ± 0.1 | 26.6 ± 0.2** | 21.8 ± 0.2** |
| | 50 | 47.3 ± 1.0** | 63.6 ± 0.3** | 28.3 ± 0.3** |

**P<0.05 represents significant difference and **P<0.01 represents extreme significant difference.*

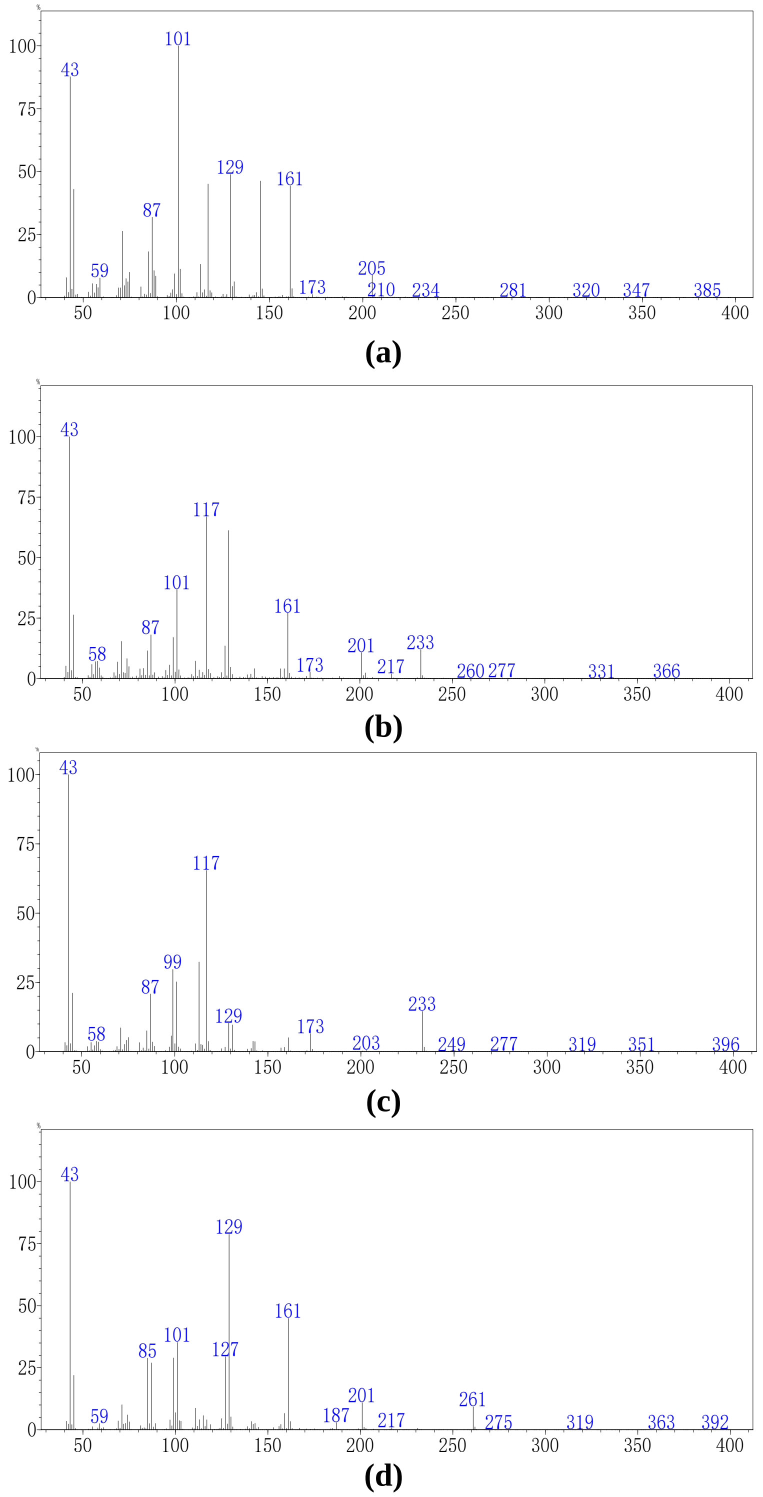


**Figure S1.** Mass spectra scanning of glycoside linkages of Man*p*-(1→ **(a)**, →3)-Man*p*-(1→ **(b)**, →4)-Glc*p*-(1→ **(c)** and →2,3)-Man*p*-(1→ **(d)**.

**Abbreviations**

*Auricularia auricula* polysaccharide, AAP; adenosine diphosphate, ADP; activated partial thromboplastin time, APTT; antithrombin III, AT-III; enzyme-linked immunosorbent assay, Elisa; endothelial nitric oxide synthases, eNOs; endothelin-1, ET-1; hematoxylin and eosin, H&E; high molecular weight kininogen, HMWK; inhibition rate of thrombus relative length, IR; protein C, PC; prostacyclin, PGI2; plasminogen, PLG; prothrombin time, PT; relative average tail length, RATL; tissue factor pathway inhibitor, TFPI; thrombin time, TT; thromboxane A2, TXA2; thromboxane B2, TXB2.
